# A prospective study of XELOX plus bevacizumab as first-line therapy in Japanese patients with metastatic colorectal cancer (KSCC 0902)

**DOI:** 10.1007/s10147-015-0895-3

**Published:** 2015-09-04

**Authors:** Yutaka Ogata, Mototsugu Shimokawa, Takaho Tanaka, Yasunori Emi, Eiji Oki, Hiroshi Saeki, Noriaki Sadanaga, Tetsuya Kusumoto, Tetsuo Touyama, Masami Kimura, Hideo Baba, Yoshito Akagi, Kazuo Shirouzu, Yoshihiko Maehara, Kyushu Study Group of Clinical Cancer (KSCC)

**Affiliations:** Department of Surgery, Kurume University Medical Center, Kurume, Japan; Department of Cancer Information Research, National Hospital Organization Kyushu Cancer Center, Fukuoka, Japan; Department of Surgery, Social Insurance Tagawa Hospital, Tagawa, Japan; Department of Surgery, Saiseikai Fukuoka General Hospital, Fukuoka, Japan; Department of Surgery and Science, Graduate School of Medical Sciences, Kyushu University, 3-1-1 Maidashi, Higashi-ku, Fukuoka, 812-8582 Japan; Department of Surgery, Nakagami Hospital, Okinawa, Japan; Department of Surgery, JCHO Hitoyoshi Medical Center, Hitoyoshi, Japan; Department of Gastroenterological Surgery, Graduate School of Medical Sciences, Kumamoto University, Kumamoto, Japan; Department of Surgery, Faculty of Medicine, Kurume University, Kurume, Japan

**Keywords:** Bevacizumab, First-line chemotherapy, Metastatic colorectal cancer, Multicenter single-arm open-label prospective clinical trial, XELOX

## Abstract

**Background:**

This study was designed to evaluate the efficacy and safety of XELOX plus bevacizumab in a Japanese metastatic colorectal cancer population that included elderly patients.

**Methods:**

This was a multicenter, single-arm, open-label prospective study. The major inclusion criteria were previously untreated metastatic colorectal cancer, presence of measurable lesions, age ≥20 years; Eastern Cooperative Oncology Group performance status of 0–2, and adequate organ function. Patients received bevacizumab (7.5 mg/kg on day 1) and XELOX (130 mg/m^2^ oxaliplatin on day 1 plus 1,000 mg/m^2^ capecitabine b.i.d. on days 1–14) every 3 weeks. The primary endpoint was confirmed objective response rate.

**Results:**

The study included 47 patients (male/female 30/17; median age 69 years; age range 38–81 years with 10 patients ≥75 years; PS 0/1/2, 40/5/2) enrolled between May 2010 and March 2011. Responses were assessed in 46 eligible patients. The objective response rate was 52.2 % (95 % confidence interval [CI] 37.0–67.1). The median progression-free survival and overall survival were 10.0 months (95 % CI 7.8–12.3) and 34.6 months (95 % CI 19.9–not estimable), respectively. Frequently encountered grade 3 and 4 adverse events in this study were aspartate aminotransferase elevation (23.4 %), alanine aminotransferase elevation (21.3 %), anorexia (12.8 %), neutropenia (10.6 %), fatigue (8.5 %) and anemia (6.4 %). Grade 3 or 4 peripheral neuropathy was not observed.

**Conclusion:**

First-line treatment with XELOX plus bevacizumab showed a promising response rate and an acceptable tolerability profile in the clinical practice of Japanese metastatic colorectal cancer patients that included elderly patients.

**Registry:**

UMIN-CTR, ID number: UMIN000003915, URL:https://upload.umin.ac.jp/cgi-open-bin/ctr/ctr.cgi?function=brows&action=brows&type=summary&recptno=R000004706&language=E

## Introduction

Colorectal cancer (CRC) is the second most common type of cancer and the third most common cause of cancer mortality in Japan [1]. Surgical removal of metastatic CRC (mCRC) is usually difficult, making chemotherapy the first choice for treatment [2].

FOLFOX4, a bi-weekly regimen of intravenous bolus and infusional 5-fluorouracil/leucovorin (5-FU/LV) plus oxaliplatin, is widely used in patients with previously untreated mCRC [3]. Capecitabine, an oral fluoropyrimidine, has shown efficacy similar to bolus 5-FU/LV as a first-line treatment for mCRC [4, 5]. Oral fluoropyrimidines can replace the intravenous fluoropyrimidine component of combination regimens. Capecitabine and a 3-week dose of oxaliplatin (the XELOX regimen) have also been shown to have efficacy similar to 5-FU/LV plus oxaliplatin (FOLFOX4 or FOLFOX6) for first-line treatment of mCRC patients [6, 7]. A pivotal phase III study (NO16966) reported that adding bevacizumab to oxaliplatin-based chemotherapy significantly improved progression-free survival (PFS) by 1.4 months when used as first-line treatment for mCRC [8]. XELOX plus bevacizumab is an effective treatment strategy with a manageable tolerability profile in Japanese patients with mCRC [9]. However, there are no prospective data in clinical practice concerning XELOX plus bevacizumab in Japan. The present study was designed to evaluate the efficacy and safety of XELOX plus bevacizumab in a Japanese mCRC population that included elderly patients.

## Patients and methods

### Study design

This was a prospective, multicenter, single-arm, open-label study that was conducted to evaluate the efficacy and safety of the commonly used doses of XELOX plus bevacizumab in a Japanese mCRC population that included elderly patients. The primary endpoint was the objective response rate (ORR), and the secondary endpoints were PFS, overall survival (OS), and safety.

### Eligibility

Patients with histologically proven, unresectable, advanced CRC or mCRC that was previously untreated were eligible for this study if they met the following criteria—measurable lesions based on Response Evaluation Criteria in Solid Tumors (RECIST), version 1.1 [10]; age ≥20 years; Eastern Cooperative Oncology Group (ECOG) performance status (PS) ≤2; life expectancy ≥3 months; no prior systemic chemotherapy for mCRC; no progression within 6 months of completion of adjuvant chemotherapy; adequate bone marrow function (neutrophil count ≥1,500/mm^3^, platelet count ≥100,000/mm^3^, and hemoglobin ≥9.0 g/dL); adequate hepatic function [total bilirubin ≤2.0 mg/dL, aspartate aminotransferase (AST) and alanine aminotransferase (ALT) ≤2.5 or 5 (in cases with liver metastases) times the institutional upper limit of normal]; adequate renal function (creatinine ≤1.5 mg/dL and protein urea ≤ grade 1); and written informed consent before enrollment in the study. The exclusion criteria included brain metastases; clinically significant ascites and pleural effusion; major surgery, open biopsy, or significant traumatic injury within 4 weeks before enrollment; fine-needle aspiration biopsy or central venous line placement within 1 week before enrollment; bleeding diathesis or coagulopathy; non-healing bone fracture; diarrhea grade ≥2; uncontrolled hypertension or peptic ulcer; clinically significant cardiovascular disease; daily treatment with high-dose aspirin (≥325 mg/day) or non-steroidal anti-inflammatory medications; immune suppressive or steroidal medications; and peripheral neuropathy grade ≥1.

### Treatment schedule

XELOX treatment included a 2-h intravenous infusion of 130 mg/m^2^ oxaliplatin (Yakult Honsha Co., Ltd., Tokyo, Japan) on day 1 plus 1,000 mg/m^2^ oral capecitabine (Chugai Pharmaceutical Co., Ltd., Tokyo, Japan) twice daily for 2 weeks of a 3-week cycle. The first dose of capecitabine was administered in the evening on day 1, and the last dose was given in the morning on day 15. Patients received 7.5 mg/kg bevacizumab (Chugai Pharmaceutical Co., Ltd.) as a 30–90-min intravenous infusion before oxaliplatin treatment on day 1 of the 3-week cycle. Treatment continued until disease progression, intolerable adverse events, or withdrawal of consent.

Treatment was interrupted if grade 2–4 toxicities were observed, and the delay continued until recovery in patients with neutrophil counts <1,500/mm^3^, febrile neutropenia, platelet counts <75,000/mm^3^, or significant persistent non-hematological toxicities. Oxaliplatin was skipped if grade ≥2 neurotoxicity was observed and bevacizumab was skipped if grade ≥2 protein urea was observed. Doses were modified based on hematological parameters and the degree of non-hematological toxicities. The capecitabine dose was reduced to 800 mg/m^2^ (or further to 600 mg/m^2^) if patients experienced grade 3 or 4 diarrhea, stomatitis, nausea or vomiting, anorexia, dermatitis, grade 4 neutropenia, or grade 3 or 4 thrombocytopenia. Oxaliplatin was also reduced to 100 mg/m^2^ (or further to 85 mg/m^2^) for all of the above conditions except for dermatitis, and was also reduced in cases of persistent (≥15 days) grade 2 neurotoxicity or temporary (8–14 days) grade 3 neurotoxicity. In patients with persistent (≥15 days) grade 3 neurotoxicity or temporary grade 4 neurotoxicity, oxaliplatin was omitted from the regimen. No dose modifications were performed for bevacizumab. This treatment plan was almost identical to that of the study NO16966 [8].

If oxaliplatin and/or bevacizumab were discontinued, treatment with the remaining components could be continued. For example, capecitabine could be administered with or without bevacizumab after discontinuation of oxaliplatin, and XELOX or capecitabine could be given after discontinuation of bevacizumab. However, continuation of oxaliplatin or bevacizumab without capecitabine was not permitted.

### Efficacy and safety evaluation

Computed tomography scans were performed to assess tumors, starting within 4 weeks prior to study registration and repeated every 6 weeks. The investigators evaluated response rates according to RECIST, version 1.1 [10]. An independent review committee (IRC) confirmed the tumor responses. PFS was defined as the time from the date of registration to the date that disease progression was first confirmed, as determined by the IRC, or to the date of death from any cause. Data were censored at the last tumor assessment if a patient withdrew before progression was observed. OS was defined as the time from the date of registration to death. Treatment continued until disease progression, unacceptable toxicity, or patient withdrawal. All eligible patients were included in the response and survival analyses (*N* = 46).

Adverse events were assessed for all enrolled patients (*N* = 47) according to the Common Terminology Criteria for Adverse Events, version 4.0.

### Relative dose intensity

Relative dose intensity can be decreased by reducing, delaying or skipping the chemotherapy dose and was calculated according to the following equation: [total actual administered dose/actual administration period (final day of the treatment course − day of the administration + 1 days)/total planned dose/planned administration period (21 days)] × 100.

### Statistical consideration

We examined whether the combination of XELOX plus bevacizumab could achieve a higher ORR than other chemotherapy regimens, as has been observed in other countries, in Japanese patients. In view of previous studies, we assumed a threshold ORR of 30 % and an expected value of at least 50 %. Under these assumptions, we determined that 39 patients were needed to provide a one-sided alpha of 0.05 and 80 % power. Factoring in a 5 % dropout rate and the possibility of ineligible patients, we set a target sample size of 41 patients. Registration was scheduled to continue for 12 months, and we planned to follow-up the patients for 36 months after the last registration. The 95 % CIs for the response rates were estimated by the Clopper−Pearson exact method. The PFS and OS curves were estimated by the Kaplan–Meier method, and their CIs were estimated using the Brookmeyer and Crowley method. All statistical analyses were performed using the SAS for Windows, release 9.3 (SAS Institute, Cary, NC, USA).

### Ethics

The ethical, medical, and scientific aspects of the study were reviewed and approved by the ethics committee of each participating institution in the UMIN clinical trials registry (UMIN000003915). The study was conducted in accordance with the Declaration of Helsinki of 1975, revised in 2000.

## Results

### Patient characteristics

A total of 47 patients were enrolled in the study between May 2010 and March 2011. One patient did not meet the eligibility criteria. Therefore, ORR, OS, and PFS were evaluated in 46 patients, while toxicity was evaluated in 47 patients treated with XELOX plus bevacizumab.

The characteristics of the 47 patients are described in Table 1. The population included 30 male and 17 female patients with a median age of 69 years (range 38–81). Twenty of the 47 patients (42 %) were ≥70 years old, and 10 patients (21 %) were ≥75 years old. The ECOG PS was 0 in 40 patients (85 %), 1 in 5 patients (11 %), and 2 in 2 patients (4 %). Affected organs were the liver in 35 patients (75 %), the lung in 10 patients (21 %), the lymph nodes in 16 patients (34 %), and the peritoneum in 7 patients (15 %). The liver was the most common site of metastasis.

**Table 1:** Patient characteristics (*N* = 47)

Factor	Characteristic	*N*
Sex	Male	30 (64 %)
	Female	17 (36 %)
Age	Median	69
	Range	38–81
Performance status (ECOG)	0	40 (85 %)
	1	5 (11 %)
	2	2 (4 %)
Primary tumor histology	Well-differentiated adenocarcinoma	7 (15 %)
	Moderately differentiated adenocarcinoma	23 (49 %)
	Poorly differentiated adenocarcinoma	6 (13 %)
	Other	2 (4 %)
	Unknown	9 (19 %)
Affected organs	Liver	35 (74 %)
	Lung	10 (21 %)
	Lymph nodes	16 (34 %)
	Peritoneum	7 (15 %)
	Local recurrence	3 (6 %)
	Other	5 (11 %)

*ECOG* Eastern Cooperative Oncology Group

### Treatment duration

The median duration of treatment was 5.0 months (range 0.7–20.0) with a median of 6.0 treatment cycles (range 1–28). XELOX plus bevacizumab combination therapy was administered for a median of 5 cycles (range 1–16). After discontinuing oxaliplatin, 3 patients (6.4 %) continued with capecitabine and bevacizumab combination therapy and a received a median of 5 cycles (range 3–20). A total of 22 patients (46.8 %) received XELOX therapy for a median of 1 cycle (range 1–4) during permanent or temporary discontinuation of bevacizumab.

Based on the planned dose intensities of 1,000 mg/m^2^ capecitabine twice daily for 2 weeks of a 3-week cycle, 130 mg/m^2^ oxaliplatin per 3-week cycle, and 7.5 mg/kg bevacizumab per 3-week cycle, the median relative dose intensities of oxaliplatin and bevacizumab were 79.0 % (95 % CI 42.4–98.1) and 75.9 % (95 % CI 41.6–96.2), respectively.

### Efficacy

The results of the tumor response analysis are shown in Table 2. A complete response (CR) was observed in 1 patient (2.2 %) and a partial response (PR) was observed in 23 patients (50.0 %) giving an overall response rate (CR + PR) of 52.2 % (95 % CI 37.0–67.1). Stable disease (SD) was observed in 15 additional patients (32.6 %). Therefore, the overall disease control rate (CR + PR + SD) was 84.8 % (95 % CI 71.1–93.7). The response rate across all time points without confirmation was 67.4 % (95 % CI 52.0–80.5).

**Table 2:** Tumor responses (*N* = 46)

RECIST-confirmed	*N* (%)
CR	1 (2.2)
PR	23 (50.0)
SD	15 (32.6)
PD	2 (4.3)
NE	5 (10.9)
ORR	24 (52.2)
90 % CI	39.2–65.0
95 % CI	37.0–67.1
DCR	39 (84.8)
95 % CI	71.1–93.7

OPR = (CR + PR), DCR = (CR + PR + SD)

*CI* confidence interval, *CR* complete response, *DCR* disease control rate, *NE* not evaluable, *ORR* overall response rate, *PD* progressive disease, *PR* partial response, *RECIST* Response Evaluation Criteria in Solid Tumors, *SD* stable disease

**Fig. 1:**
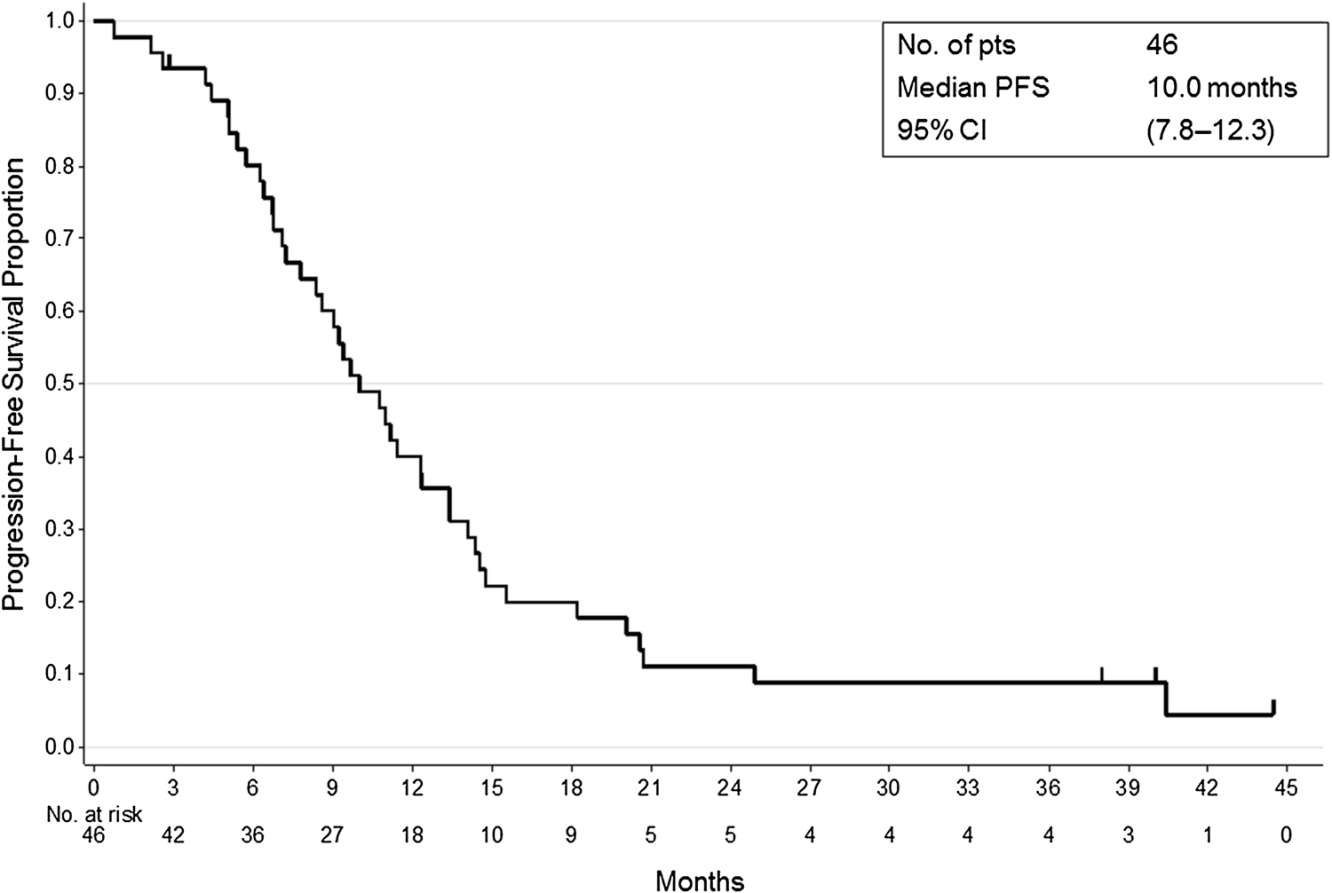
Kaplan–Meier estimate for progression-free survival (PFS). After a median follow-up time of 34.4 months, the median PFS was 10.0 months (95 % CI 7.8–12.3)

**Fig. 2:**
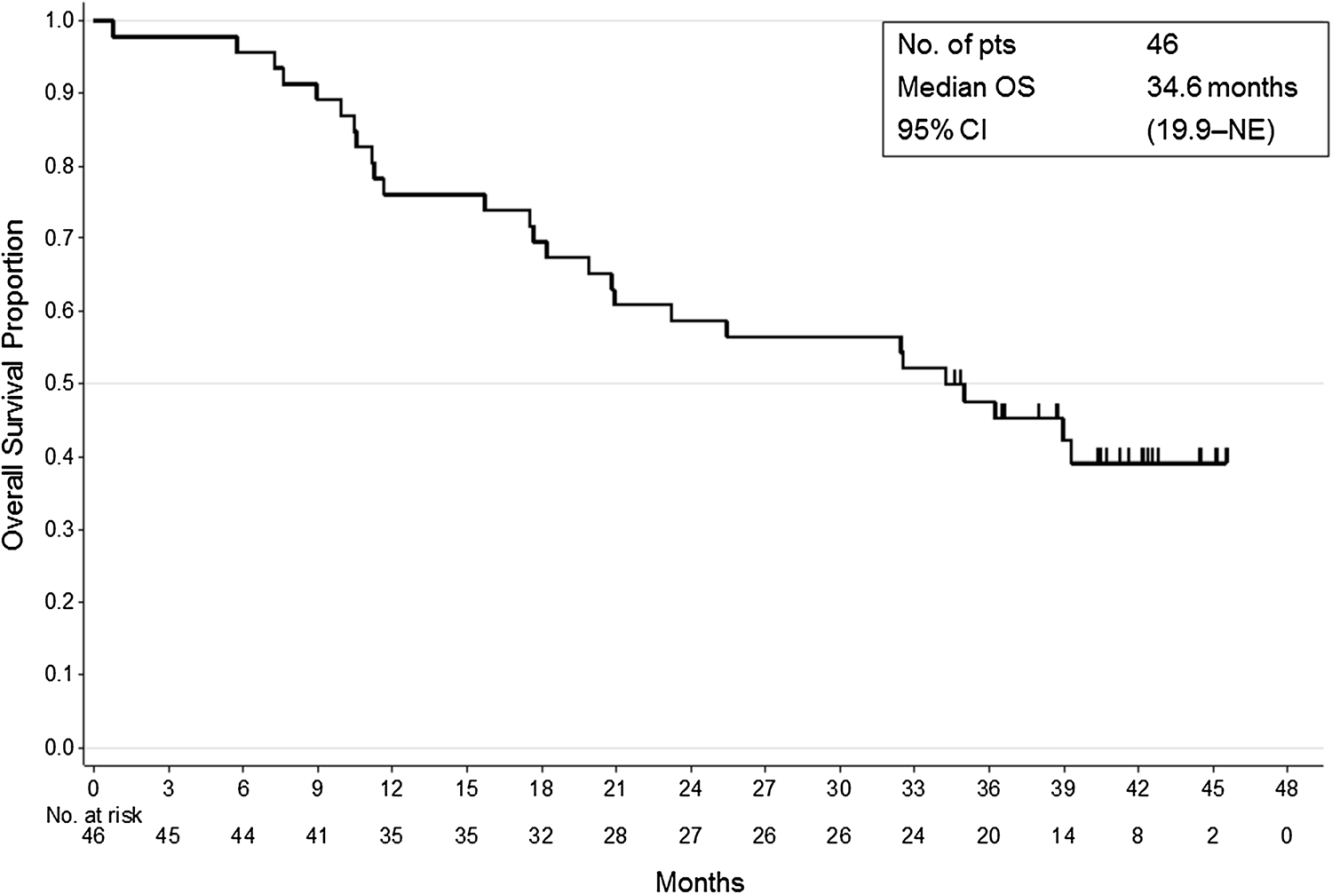
Kaplan–Meier estimate for overall survival (OS). The median OS was 34.6 months (95 % CI 19.9–not estimable)

The cut-off date for PFS and OS was April 2014. The median follow-up period was 34.4 months. Median PFS was 10.0 months (95 % CI 7.8–12.3; Fig. 1). A total of 27 of the 46 eligible patients died due to progression of advanced colorectal cancer. At the time of analysis, the median OS was 34.6 months (95 % CI 19.9–not estimable; Fig. 2).

### Safety

Toxicity data are available for 47 patients treated with a median of 6.0 chemotherapy cycles (range 1–28). Toxicities are summarized in Table 3. Frequently encountered non-hematological toxicities included peripheral neuropathy, hand-foot syndrome, skin hyperpigmentation, fatigue, and gastrointestinal adverse effects such as diarrhea. Most of the non-hematological toxicities were grade 1 or 2. Frequently encountered adverse effects included grade 3 or 4 anorexia and fatigue, which were recorded in 6 (12.8 %) and 4 (8.5 %) of the 47 patients, respectively. No grade 3 or 4 peripheral neuropathy was observed. Other grade 3 or 4 non-hematological toxicities were febrile neutropenia, nausea, hand-foot syndrome, and oral mucositis, each of which occurred in 1 patient. With regard to hematological toxicities, including laboratory disorders, frequently encountered toxicities were grade 3 or 4 neutropenia, leukopenia, thrombocytopenia, anemia, and elevation of AST and ALT, which were recorded in 5 (10.6 %), 2 (4.3 %), 2 (4.3 %), 3 (6.4 %), 11 (23.4 %), and 10 (21.3 %) of the 47 patients, respectively. Frequently encountered bevacizumab-related toxicities were hypertension in 22 patients (46.8 %) and proteinuria in 20 patients (47.6 % of 42 patients). Other bevacizumab-related toxicities included a thromboembolic event, gastrointestinal hemorrhage, and gastrointestinal perforation, each of which occurred in 1 patient (2.1 %).

**Table 3:** Adverse events related to treatment (*N* = 47)

Adverse event	Grade 1	Grade 2	Grade 3	Grade 4	Grade 3/4
Febrile neutropenia	0 (0.0 %)	0 (0.0 %)	1 (2.1 %)	0 (0.0 %)	1 (2.1 %)
Fatigue	17 (36.2 %)	5 (10.6 %)	4 (8.5 %)	0 (0.0 %)	4 (8.5 %)
Diarrhea	14 (29.8 %)	7 (14.9 %)	0 (0.0 %)	0 (0.0 %)	0 (0.0 %)
Nausea	15 (31.9 %)	9 (19.1 %)	1 (2.1 %)	0 (0.0 %)	1 (2.1 %)
Vomiting	8 (17.0 %)	2 (4.3 %)	0 (0.0 %)	0 (0.0 %)	0 (0.0 %)
Anorexia	16 (34.0 %)	9 (19.1 %)	6 (12.8 %)	0 (0.0 %)	6 (12.8 %)
Alopecia	1 (2.1 %)	0 (0.0 %)	0 (0.0 %)	0 (0.0 %)	0 (0.0 %)
Hand-foot syndrome	18 (38.3 %)	6 (12.8 %)	1 (2.1 %)	0 (0.0 %)	1 (2.1 %)
Oral mucositis	14 (29.8 %)	1 (2.1 %)	1 (2.1 %)	0 (0.0 %)	1 (2.1 %)
Dysgeusia	13 (27.7 %)	3 (6.4 %)	0 (0.0 %)	0 (0.0 %)	0 (0.0 %)
Skin hyperpigmentation	20 (42.6 %)	1 (2.1 %)	0 (0.0 %)	0 (0.0 %)	0 (0.0 %)
Hypersensitivity	1 (2.1 %)	0 (0.0 %)	0 (0.0 %)	0 (0.0 %)	0 (0.0 %)
Peripheral neuropathy	21 (44.7 %)	18 (38.3 %)	0 (0.0 %)	0 (0.0 %)	0 (0.0 %)
Blood bilirubin increased	10 (21.3 %)	7 (14.9 %)	1 (2.1 %)	0 (0.0 %)	1 (2.1 %)
AST increased	19 (40.4 %)	4 (8.5 %)	10 (21.3 %)	1 (2.1 %)	11 (23.4 %)
ALT increased	14 (29.8 %)	4 (8.5 %)	9 (19.1 %)	1 (2.1 %)	10 (21.3 %)
ALP increased (*N* = 45)	19 (42.2 %)	2 (4.4 %)	1 (2.2 %)	0 (0.0 %)	1 (2.2 %)
Creatinine increased	10 (21.3 %)	2 (4.3 %)	1 (2.1 %)	0 (0.0 %)	1 (2.1 %)
Leukopenia	15 (31.9 %)	14 (29.8 %)	2 (4.3 %)	0 (0.0 %)	2 (4.3 %)
Neutropenia	8 (17.0 %)	23 (48.9 %)	5 (10.6 %)	0 (0.0 %)	5 (10.6 %)
Thrombocytopenia	23 (48.9 %)	7 (14.9 %)	2 (4.3 %)	0 (0.0 %)	2 (4.3 %)
Anemia	28 (59.6 %)	12 (25.5 %)	2 (4.3 %)	1 (2.1 %)	3 (6.4 %)
Hypertension	9 (19.1 %)	11 (23.4 %)	2 (4.3 %)	0 (0.0 %)	2 (4.3 %)
Thromboembolic event	0 (0.0 %)	0 (0.0 %)	0 (0.0 %)	1 (2.1 %)	1 (2.1 %)
Proteinuria (*N* = 42)	14 (33.3 %)	6 (14.3 %)	0 (0.0 %)	0 (0.0 %)	0 (0.0 %)
Gastrointestinal hemorrhage	0 (0.0 %)	0 (0.0 %)	1 (2.1 %)	0 (0.0 %)	1 (2.1 %)
Gastrointestinal perforation	0 (0.0 %)	1 (2.1 %)	0 (0.0 %)	0 (0.0 %)	0 (0.0 %)

*ALT* alanine aminotransferase, *AST* aspartate aminotransferase, *ALT* alkaline phosphatase

A total of 15 patients (31.9 %) discontinued the protocol because of adverse events—5 patients with neurosensory toxicity, 4 patients with anorexia, 4 patients with fatigue, 2 patients with neutropenia, 2 patients with hypertension, 1 patient with gastrointestinal hemorrhage, 1 patient with diarrhea, 1 patient with a thromboembolic event, and 1 patient with hand-foot syndrome.

### Post-treatment

After treatment with XELOX plus bevacizumab, 18 patients (38.3 %) underwent surgery and 4 patients (8.5 %) received radiation therapy. Thirty-six of the 47 patients (76.6 %) were treated with second-line chemotherapy, and 21 of those 36 patients (58.3 %) received bevacizumab continuously. Two patients received combination chemotherapy with anti-epidermal growth factor receptor (EGFR) antibody as second-line treatment, and 13 patients received the anti-EGFR antibody alone or in combination with cytotoxic agents after second-line chemotherapy. Seven patients (14.9 %) were treated with best supportive care.

### Efficacy analyses according to age

A summary of efficacy according to patient age (≥70 vs <70 years) is shown in Table 4. There were no significant differences in ORR (42.1 vs 59.3 %, *p* = 0.370), PFS (9.7 vs 12.3 months, *p* = 0.179), or OS (23.2 months vs not reached, *p* = 0.069) based on patient age.

**Table 4:** Summary of treatment efficacy according to age

Responses (RECIST-confirmed)	*N*	Events	ORR (%)	95 % CI	*p* value (fisher’s exact test)
≥70 years	19	8	42.1	20.3–66.5	* 0.3695*
<70 years	27	16	59.3	38.8–77.6	

PFS	*N*	Median PFS (months)		95 % CI	*p* value (log-rank test)
≥70 years	19	9.7		6.4–11.4	*0.1788*
<70 years	27	12.3		7.2–14.5	

OS	*N*	Median OS (months)		95 % CI	*p* value (log-rank test)
≥70 years	19	23.2		11.6–38.9	* 0.0688*
<70 years	27	NE		19.9–NE	

*CI* confidence interval, *NE* not estimable, *ORR* overall response rate, *OS* overall survival, *PFS* progression-free survival, *RECIST* Response Evaluation Criteria in Solid Tumors

### Safety analyses according to age

A summary of safety according to age (≥70 vs <70 years) is shown in Table 5. The toxicity profile in elderly patients was similar to that in younger patients, with the exceptions of neutropenia and leukopenia; elderly patients had lower rates of neutropenia and leukopenia than younger patients.

**Table 5:** Summary of safety according to age

	≥70 years (*N* = 20)		*p* value	<70 years (*N* = 27)	
	All grades	Grade 3/4	All grades	All grades	Grade 3/4
Leukopenia	10 (50.0 %)	1 (5.0 %)	0.0650	21 (77.8 %)	1 (3.7 %)
Neutropenia	11 (55.0 %)	3 (15.0 %)	0.0044	25 (92.6 %)	2 (7.4 %)
Thrombocytopenia	13 (65.0 %)	1 (5.0 %)	0.7583	19 (70.4 %)	1 (3.7 %)
Anemia	20 (100.0 %)	2 (10.0 %)	0.1256	23 (85.2 %)	1 (3.7 %)
Fatigue	12 (60.0 %)	2 (10.0 %)	0.7674	14 (51.9 %)	2 (7.4 %)
Diarrhea	10 (50.0 %)	0 (0.0 %)	0.5661	11 (40.7 %)	0 (0.0 %)
Anorexia	14 (70.0 %)	4 (20.0 %)	0.7583	17 (63.0 %)	2 (7.4 %)
Nausea	10 (50.0 %)	1 (5.0 %)	0.7729	15 (55.6 %)	0 (0.0 %)

## Discussion

Previous randomized and observational trials that have included the XELOX plus bevacizumab regimen as first-line therapy have been mainly conducted in North America and Europe [8, 11, 12]. The NO16966 study [8, 13] reported longer median PFS (9.3 vs 7.4 months; hazard ratio 0.77; 95 % CI 0.6–0.94; *p* = 0.0026) and median OS (21.6 vs 18.8 months) in the XELOX plus bevacizumab arm than in the XELOX plus placebo arm in a subgroup analysis. Furthermore, another phase III trial (CAIRO2) reported an ORR of 50.0 %, a median PFS of 10.7 months, and a median OS of 20.3 months in patients receiving XELOX plus bevacizumab [11]. In this prospective clinical study of Japanese mCRC patients that included elderly patients, XELOX plus bevacizumab achieved similar efficacy and safety as previous pivotal phase III studies of XELOX plus bevacizumab [8, 13], even in elderly patients. The ORR was 52.2 % (95 % CI 37.0–67.1). The median PFS and median OS were 10.0 months (95 % CI 7.8–13.8) and 34.6 months (95 % CI 19.9–not estimable), respectively. These outcomes are also similar to the favorable results from the phase I/II Japanese clinical trial of XELOX plus bevacizumab in mCRC patients (median PFS 11.0 months; median OS 27.4 months) [9] and to the results from a retrospective analysis of XELOX plus bevacizumab in clinical practice for Japanese mCRC patients (median PFS 290 days; median OS 816 days) [14].

Recent phase III trials as first-line chemotherapy plus biologics for mCRC patients yielded a favorable median OS of approximately 30 months [15, 16]. The OS in the present study was also favorable (34.6 months), as was the phase I/II Japanese clinical trial [9]. One reason for the favorable OS in our study may be that most patients received sequential therapy such as chemotherapy, radiotherapy, and surgery. A total of 21 patients (58.3 %) received bevacizumab-containing regimens as second-line chemotherapy in our study. Therefore, improved OS might partially depend on the sequential use of bevacizumab beyond disease progression [17], as it reportedly resulted in statistically significant increases in OS as second-line therapy in combination with irinotecan or oxaliplatin-based regimens. Phase III studies of another class of biologics, anti-EGFR antibodies (cetuximab and panitumumab), showed improved OS in patients with mCRC for whom other treatments had failed [18, 19]. In our study, 2 patients received anti-EGFR antibody-containing regimens as second-line therapy, and 13 patients received an anti-EGFR antibody alone or in combination with cytotoxic agents after second-line therapy. The anti-EGFR antibodies may have partially contributed to the improvement in OS.

The toxicity profiles in our study were generally predictable and manageable. Elevated AST, increased ALT, and neutropenia were the most common grade 3 and 4 hematologic toxicities; these events were observed in 23.4, 21.3, and 10.6 % of the patients, respectively. Anorexia and fatigue were the most common grade 3 and 4 non-hematologic toxicities; these events occurred in 12.8 and 8.5 % of the patients, respectively. The incidence of other grade 3 and 4 toxicities including neurotoxicity was extremely low. The incidences of grade 3 or 4 neurotoxicity were reportedly 15 [14] and 17 % [9] in the Japanese trials with XELOX plus bevacizumab. In a number of trials of oxaliplatin-based therapies, neurotoxicity was the most frequently encountered adverse event that led to discontinuation of treatment. In our study, no grade 3 or 4 neurotoxicity was observed, because an administration criterion of oxaliplatin was grade 0 or 1 neurotoxicity. However, the neurotoxicity led to discontinuation of treatment in 5 of 47 patients (10.6 %). The overall rate of protocol discontinuation due to adverse events was 31.9 %, which might be acceptable.

The treatment schedule and doses selected for our study were identical to those in the NO16966 study [8, 13]. The median relative dose intensities with consideration of both dose reduction and treatment delay/skip in this study were acceptable. These could not be accurately compared to those in the XELOX plus bevacizumab arm of the NO16966 study (ratio of dose received to dose planned), because there might be a difference in calculation method between the studies.

It is notable that 20 of the 47 patients enrolled in our trial were ≥70 years. Previous studies have established the efficacy and safety of combination chemotherapy in elderly patients [20–22]. The addition of bevacizumab to chemotherapy in geriatric populations has also been shown to be effective in observational cohort studies, subgroup analyses, and pooled analyses of cohorts from other randomized trials [23–25]. A phase II study reported that an alternative XELOX plus bevacizumab (AXELOX) regimen was an effective and safe combination for elderly patients (age >70 years) with mCRC [26]. AXELOX treatment includes intravenous infusion of 5 mg/kg bevacizumab, 85 mg/m^2^ oxaliplatin on day 1, and 750 mg/m^2^ oral capecitabine twice daily for 1 week of a 2-week cycle. The AXELOX regimen achieved favorable efficacy (ORR 46.8 %; PFS 7.9 months; OS 20.1 months) and low toxicity (incidence of all grade 3 or 4 adverse events <10 %). The low toxicity of AXELOX is thought to be attributable to the low dose intensity of capecitabine, which is 56 % of the international standard dose of XELOX plus bevacizumab used in our study. However, in our study, the efficacy and safety for elderly patients ≥70 years were favorable in a manner similar to results achieved with younger patients <70 years. Our results suggest that the international standard dose of XELOX plus bevacizumab might be effective and safe for both younger and elderly Japanese patients with mCRC. Moreover, the XELOX regimen requires only one visit per 3-week cycle for a 2- or 3-h infusion, and no portable pump is required, which may provide a marked advantage over the FOLFOX regimen in terms of patient convenience.

In conclusion, this study found that first-line XELOX plus bevacizumab showed good tolerability and efficacy in clinical practice for the treatment of mCRC in a Japanese population that included elderly patients. XELOX plus bevacizumab may be considered a routine first-line treatment option for mCRC patients.
